# Hypersensitivity to intravenous succinate corticosteroids in patients with nonsteroidal anti-inflammatory drug-exacerbated respiratory disease

**DOI:** 10.3389/falgy.2023.1145809

**Published:** 2023-11-10

**Authors:** Masami Taniguchi, Atsuhiko Sato, Haruhisa Mita

**Affiliations:** ^1^ Department of Respiratory Medicine, Hamamatsu University School of Medicine, Hamamatsu, Japan; ^2^ Clinical Research Center for Allergy and Rheumatology, National Hospital Organization Sagamihara National Hospital, Sagamihara, Japan

**Keywords:** corticosteroids, N-ERD, succinate, hypersensitivity, AERD (aspirin-exacerbated respiratory disease)

## Abstract

Although there are many case reports of asthma exacerbations with intravenous corticosteroids, especially hydrocortisone succinate, in nonsteroidal anti-inflammatory drug-exacerbated respiratory disease (N-ERD), the frequency and mechanism remain unclear. We hypothesized that N-ERD patients are potentially hypersensitive to succinates, especially succinate corticosteroids, based on the results of previous provocation studies and considered specific mechanisms. The objective of this study was to determine the frequency and mechanism of succinate corticosteroids hypersensitivity in patients with N-ERD. Eleven patients with stable, moderate to severe N-ERD were tested with hydrocortisone sodium succinate (HCs), hydrocortisone sodium phosphate (HCp), methylprednisolone sodium succinate (MPSLs), prednisolone sodium succinate (PSLs), and chloramphenicol sodium succinate (CPs, without a steroidal chemical structure) at doses below the normal dose through intravenous administration using a single-blind test. As a comparison, seven patients with aspirin-tolerant asthma (ATA) also underwent an intravenous provocation test of HCs. The positive intravenous provocation test rates of HCs 100–500 mg, HCp 500 mg, MPSLs 80 mg, PSLs 20 mg, and CPs 500 mg in N-ERD patients were 82% (9/11), 9% (1/11), 50% (5/10), 33% (1/3), and 86% (6/7), respectively. Most positive reactions began with a severe cough within 5 min of intravenous injection. The course of these hypersensitivity symptoms differed from those seen with the usual aspirin challenge test. The HCs 100–500 mg intravenous test was negative in all seven patients with ATA. In conclusion, patients with N-ERD have high rates of potential hypersensitivity to the succinate ester structure, which is not linked to the corticosteroid structure, but to the succinate ester structure. We hypothesized that the mechanism of hypersensitivity observed during rapid intravenous administration of succinate corticosteroids is mast cell activation via succinate receptor stimulation, rather than due to the corticosteroid itself.

## Introduction

Nonsteroidal anti-inflammatory drug-exacerbated respiratory disease (N-ERD) is an acquired, non-genetic intolerance to aspirin and other cyclooxygenase 1 (COX-1)-inhibiting NSAIDs that causes nasal obstruction and asthma exacerbation (1–7). Since the 2000s, NSAIDs, including selective COX-2 inhibitors such as celecoxib, have been found to be safe to use in patients with N-ERD (8, 9). Furthermore, it is widely known that NSAIDs with stronger COX-1 inhibition are more likely to induce asthma attacks; thus, the main feature of N-ERD is now considered to be COX-1 inhibitor intolerance (1–3, 5).

It has been historically reported that asthma exacerbation in patients with N-ERD can also be induced by alcohol and various additives (10–13). In addition, many reports of asthma exacerbation by systemic corticosteroids, especially intravenous corticosteroids, have been reported in patients with N-ERD (14–23). However, the causative agent and mechanism remain unknown.

Corticosteroids are insoluble in water. Therefore, for intravenous administration, corticosteroids are typically formulated as water-soluble esters: succinate or phosphate ester. In contrast, corticosteroids for oral use do not need to be water-soluble and therefore, have nearly the same chemical structure as endogenous corticosteroids. Previous reports of corticosteroid hypersensitivity in N-ERD had implicated the phosphate type as the cause in a few cases and the succinate type as the cause in most cases (14–18, 21–23). Thus, we hypothesized that N-ERD patients could be hypersensitive to the succinate ester structure, rather than to the corticosteroids themselves.

### Presentation of a typical case from real-world settings

The patient was a 39-year-old Japanese female with a history of asthma exacerbation with aspirin from 3 years ago. She visited our hospital because her asthma attacks did not improve with beta-agonist inhalation at home. After a saline infusion, 100 mg of HCs (Solu-Cortef®) dissolved in 20 ml saline without additives was given intravenously over several minutes. A few minutes later, she developed a marked asthma exacerbation with severe cough and cyanosis and finally recovered after 1 h of oxygen administration, intramuscular injection of adrenaline, and intravenous theophylline infusion. No skin rash or hypotension was observed during the course of hypersensitivity symptoms, and her asthma exacerbation was judged to be due to the intravenous hydrocortisone sodium succinate (HCs) preparation.

Later, for the purpose of studying available intravenous steroids, a single-blind, loading study was performed four times every 2–4 weeks with oral cortisol and three intravenous corticosteroid formulations, with the patient's consent. Intravenous infusion was performed over 5 min. The results showed that prednisolone sodium succinate (PSLs) 10 mg and methylprednisolone sodium succinate (MPSLs) 20 mg decreased forced expiratory volume in 1 s (FEV1) by 31% and 35%, respectively, and induced severe coughing and wheezing. A subsequent intravenous provocation test with hydrocortisone sodium phosphate (HCp), starting at 10 mg and going up to a maximum of 100 mg, did not decrease FEV1 and did not induce coughing. Based on these results and an analysis of reported cases in the literature, we hypothesized that N-ERD is potentially hypersensitive to succinate steroids such as HCs.

## Method

The present study was conducted using historical records from the 1980s of provocation test results in patients with N-ERD that have not been published as papers (17). All eligible patients were given a detailed description of the study before obtaining their verbal consent. Eleven patients with stable, moderate to severe N-ERD without any history of corticosteroid hypersensitivity underwent the following intravenous provocation tests of several types of corticosteroids and CPs at 2–4-week intervals in a single-blind fashion. Asthma severity was determined by Global Initiative for Asthma (GINA) classification (24). Aspirin hypersensitivity in all patients was confirmed by a standard oral aspirin challenge test (25, 26) or by an intravenous lysin-aspirin challenge test, which we have previously reported (27, 28). In addition, seven ATA patients were subjected to two separate, induced-challenge tests with 100 mg HCs and 500 mg HCs. The provocation tests were performed only in patients with FEV1% > 70% after discontinuation of inhaled beta agonist during a stable period. The following five drugs were used intravenously over a 5-min period in the provocation tests. The loading dose was set at a dose commonly used in clinical practice.

(1) Saline
(2) HCs (Solu-Cortef®) 100 mg, 500 mg
(3) HCp (Hydrocortone®) 500 mg
(4) MPSLs (Solu-Medrol®) 80 mg
(5) PSLs (Predonine® for injection) 20 mg
(6) CPs (Chloromycetin® for injection) 500 mg

The reasons for selecting CPs for the provocation test are as follows: CPs is a classic antibiotic that has been used for many years and is the only intravenous succinate available in Japan without a steroid structure and without additives. Furthermore, a single dose of 1,000–2,000 mg of CPs has been reported to cause few adverse reactions, including anaphylaxis. Positive results were defined as a decrease by at least 20% of the pre-provocation value in FEV1 after 30 min of injection. In addition, the presence of severe coughing with no measurable pulmonary function was also considered a positive result. The severity of the induced asthma attack was classified according to the GINA guidelines as follows: mild: able to lie in a supine position and no decrease in saturation of percutaneous oxygen (SpO_2_); severe: SpO_2_ below 90% or difficulty in speaking; moderate: other than mild and severe (24). All succinate corticosteroids and CPs were diluted in 20 ml of saline without additives. However, because HCp (Hydrocortone®) is only available as a solution containing additives such as parabens and sulfites; this commercial formulation was also used for the provocation test.

## Result

The patient characteristics are shown in Table 1: eleven patients with N-ERD and seven patients with ATA were selected, all with moderate to severe stable asthma, no daily lung function deterioration, and no problems with discontinuation of anti-asthmatic drugs in the provocation test. Except for case 1, there was no history of hypersensitivity to corticosteroids. Note that Case 1 in this Table 1 refers to the same patient as in the case presentation in the previous section.

**Table 1:** Patient characteristics.

	N-ERD (*n* = 11)	ATA (*n* = 7)	Significant difference
Age (years)	39–64	41–59	NS
Sex (M: F)	3:8	2:5	NS
Body weight	41–71 kg	45–65 kg	NS
Smoker	0/11	1/7	NS
CRSwNP	11/11	1/7	*p* < 0.001
Asthma severity	Severe: 5 moderate: 6	Severe: 3 moderate: 4	NS
ICS (BDP equivalent)	800 μg/day	800 μg/day	NS
OCS (PSL)	0–5 mg/day	0–5 mg/day	NS
Serum IgE	20–451 IU/ml	35–755 IU/ml	NS
Eosinophilia	325–551/mm^3^	275–390/mm^3^	NS
FEV1%	75.5%–83.1%	78.8%–82.3%	NS

N-ERD, nonsteroidal anti-inflammatory drug-exacerbated respiratory disease; ATA, aspirin-tolerant asthma; NS, not significant; CRSwNP, chronic rhinosinusitis with nasal polyposis; OCS, oral corticosteroid; PSL, prednisolone; ICS, inhaled corticosteroid; BDP, beclomethasone dipropionate; FEV1%, forced expiratory volume % in 1 s.

Aspirin sensitivity was confirmed in all subjects by systemic aspirin provocation test prior to this study. CRSwNP was determined based on CT images and the otolaryngologist's judgment. Eosinophil counts in peripheral blood. Asthma severity was determined according to GINA guidelines (24).

**Figure 1:**
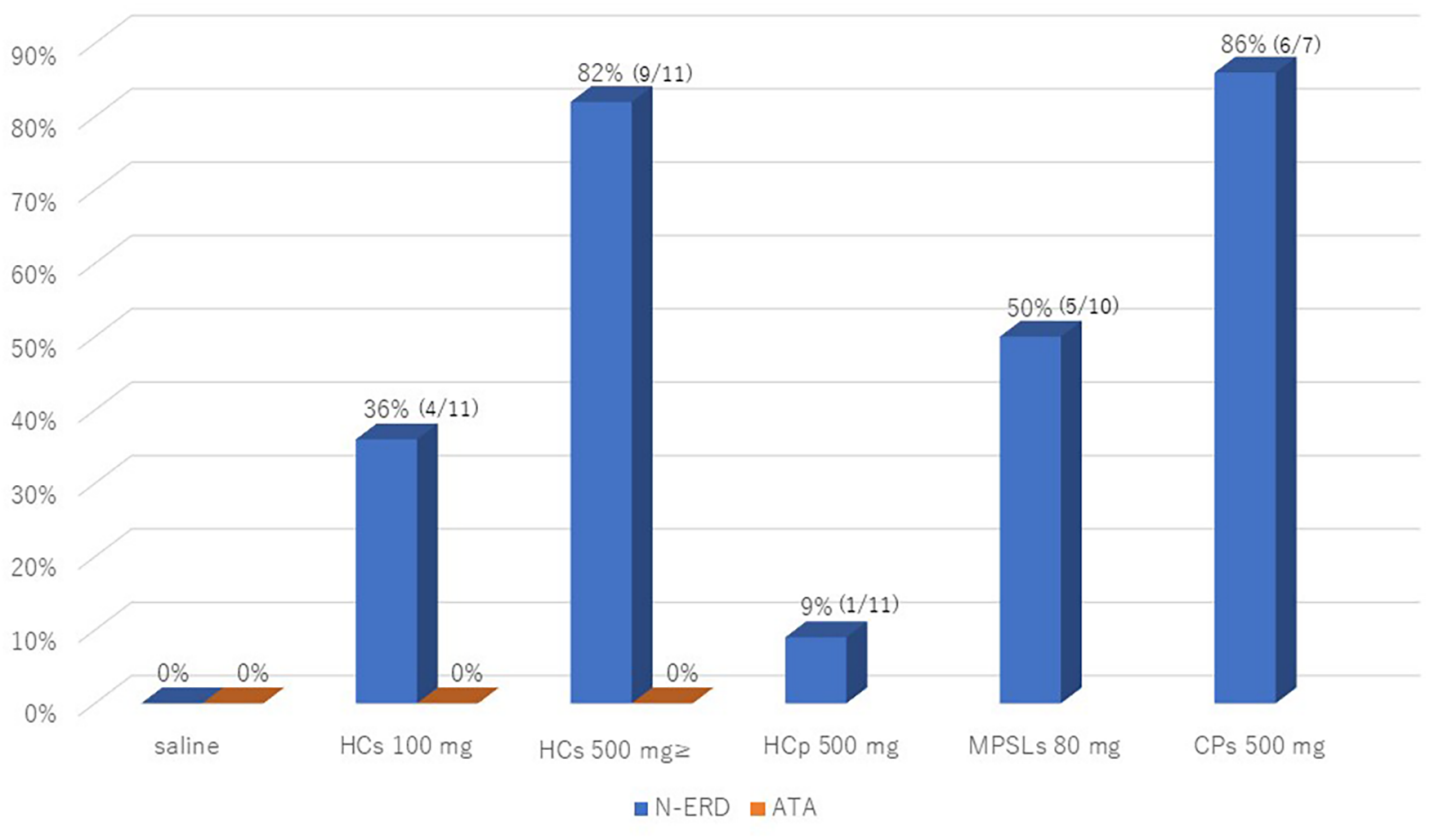
The positive intravenous provocation test rates of saline and HCs 100/500 mg in N-ERD and ATA patients, and positive intravenous test rates of HCp 500 mg, MPSLs 80 mg, and CPs 500 mg in N-ERD patients. N-ERD, nonsteroidal anti-inflammatory drug-exacerbated respiratory disease; ATA, aspirin-tolerant asthma; HCs, hydrocortisone sodium succinate; HCp, hydrocortisone sodium phosphate; MPSLs, methylprednisolone sodium succinate; CPs, chloramphenicol sodium succinate.

The results of the administration of various intravenous steroids and intravenous chloramphenicol provocation tests in N-ERD are shown in Table 2 and Figure 1. The placebo (saline 20 ml) infusion test gave negative results in all 11 N-ERD subjects. In the 11 N-ERD patients, the decrease in FEV1 after 100 mg of intravenous HCs ranged from −8.1% to −35.5% (median 13.1%) from the preprovocation value, with 4/11 positive test results showing a decrease in FEV1 of 20% or more. In an intravenous study of 500 mg of HCs in 7 NERD patients who were negative on 100 mg of HCs, FEV1 decreased from 10.1% to 51.5% (median 31.3%) from preprovocation level and was positive in 5/7 patients. Many of the positive cases had severe coughing requiring beta-agonist inhalation and small doses of adrenaline therapy. Thus, 9/11 (82%) patients were positive for less than 500 mg of HCs in N-ERD. In all cases, although a severe cough was induced within 5 min after injection, no skin rash was observed. MPSLs 80 mg and PSLs 20 mg were additionally tested in some patients with N-ERD and were positive in 5/10 (50%) and 1/3 (33%) cases, respectively. The induced symptoms were similar to those observed with HCs. MPSLs and PSLs provocation tests were not attempted in all 11 patients for the sake of patient convenience. In the HCp 500 mg provocation test, only 1 of 11 patients (9%) had a mild cough with no decrease in lung function (Figure 1).

**Table 2:** Results of various intravenous corticosteroids and intravenous chloramphenicol sodium succinate provocation studies in 11 N-ERD patients.

Patients	Provocations drugs							
No.	Sex, age	Saline	HC_S_ 100 mg	HC_S_ 500 mg	HCp 500 mg	MPSL_S_ 80 mg	PSL_S_ 20 mg	CP_S_ 500 mg
1	F, 39	−	+	ND	−	+	+	+ (severe)
2	F, 61	−	+	ND	±	+	−	+ (severe)
3	F, 54	−	−	+	−	−	−	+ (severe)
4	F, 48	−	−	+	−	+	ND	+ (severe)
5	F, 41	−	−	+	−	−	ND	ND
6	F, 49	-	+	ND	−	+	ND	+
7	F, 64	−	−	+ (severe)	−	−	ND	ND
8	F, 55	−	+	ND	−	+	ND	ND
9	M, 42	−	−	−	−	−	ND	−
10	M, 51	−	−	−	−	−	ND	+
11	M, 55	−	−	+ (severe)	−	ND	ND	ND
Percentage of positive reactions (number)		0% (0/11)	36% (4/11)	71% (5/7)	9% (1/11)	50% (5/10)	33% (1/3)	86% (6/7)

N-ERD, nonsteroidal anti-inflammatory drug-exacerbated respiratory disease; HCs, hydrocortisone sodium succinate; HCp, hydrocortisone sodium phosphate; MPSLs, methylprednisolone sodium succinate; PSLs, prednisolone sodium succinate; CPs, chloramphenicol sodium succinate; F, female; M, male.

ND, not done (discontinued for patient's convenience, but CPs provocation test was discontinued due to severe symptoms).

Severe: severe cough and dyspnea developed; lung function was unmeasurable, SpO_2_ decrease and required adrenaline administration.

+, positive reaction.

−, negative reaction without symptoms.

±, only cough appeared, but no significant decrease in lung function.

A provocation study in N-ERD was also performed with CPs, an intravenous succinate without corticosteroid structure. Severe coughing and wheezing with no measurable pulmonary function were induced in 6 of 7 patients (88%) (Figure 1). Therefore, for safety reasons, we decided not to perform the CPs loading test in the remaining 4 cases. The positive symptoms in all provocation tests resolved within 2 h with the inhalation of beta bronchodilators and, in some cases, subcutaneous injection of a small amount of adrenaline. In contrast, the HCs 100–500 mg intravenous provocation test was negative in all seven patients with ATA.

## Discussion

This study demonstrates for the first time that N-ERD patients are potentially hypersensitive not only to HCs but also to succinate esters of other corticosteroids, and that similar airway symptoms are induced by CPs without steroidal structures. These results suggest that the hypersensitivity in N-ERD patients is not due to the structure of the corticosteroid, but to the succinate ester structure.

Although only one N-ERD patient showed asthma exacerbation with HCp in the present results, it was very mild and was presumed to be caused by an additive in the HCp formulation. Hypersensitivity in N-ERD patients due to sulfites and parabens in the HCp formulation (Hydrocorton®) has been reported in many cases. However, there is a lack of evidence for this relationship, and further studies are needed. On the other hand, none of the ATA patients had hypersensitivity reactions to these preparations, indicating that this succinate hypersensitivity is specific to the pathogenesis of N-ERD.

**Figure 2:**
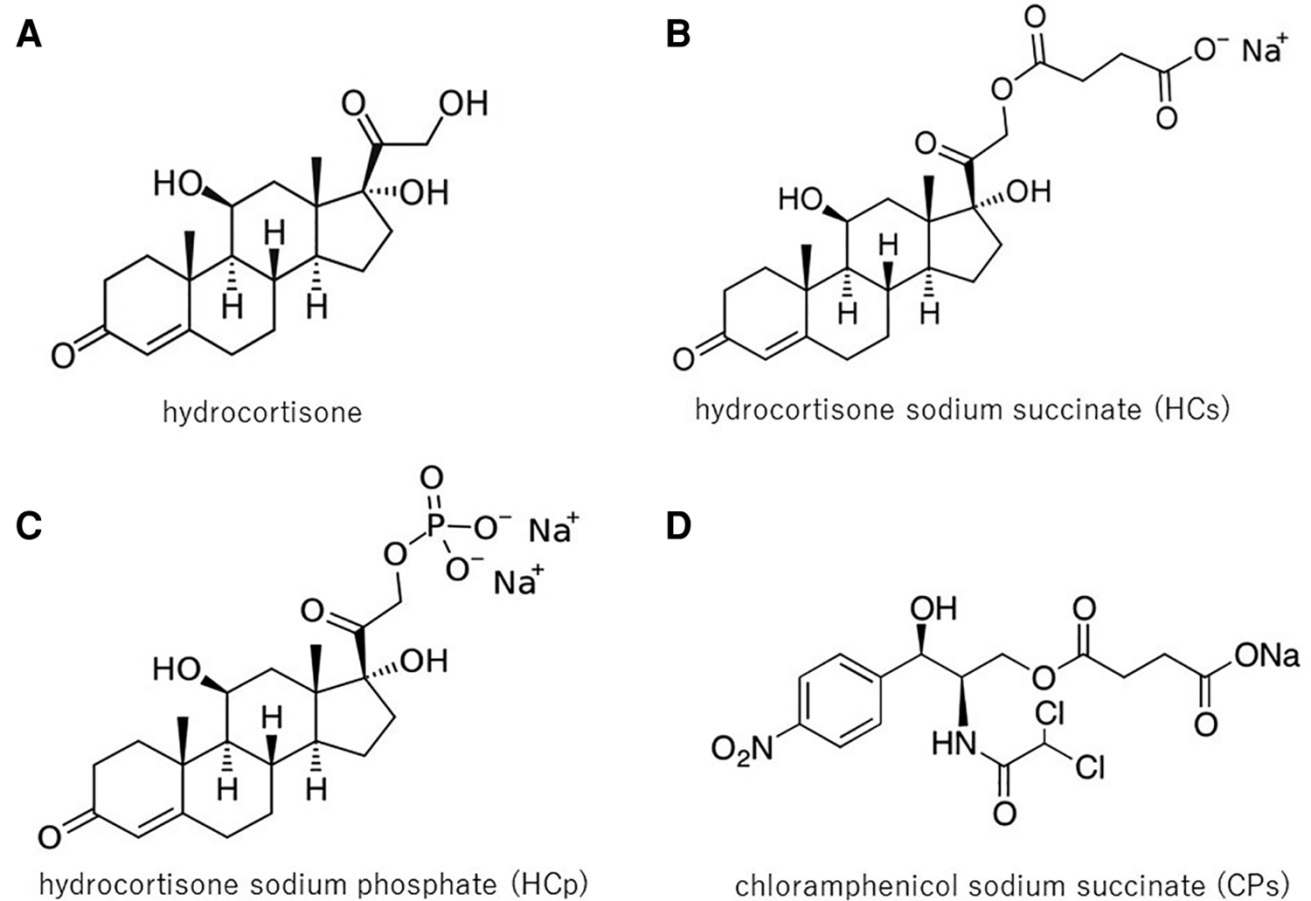
Chemical structures of HC, HCs, HCp, and CPs: HC (**A**) is insoluble in water. Therefore, for intravenous administration, corticosteroids are typically formulated as water-soluble esters: succinate or phosphate ester. N-ERD patients were hypersensitive to HCs (**B**) and CPs (**D**), but not to HC (**A**), and showed little reaction to HCp (**C**), thus indicating that hypersensitivity was not associated with the steroid structure itself. HC, hydrocortisone; HCs, hydrocortisone sodium succinate; HCp, hydrocortisone sodium phosphate; CPs, chloramphenicol sodium succinate.

Figure 2 shows the chemical structures of hydrocortone (Figure 2A) and its water-solubilized forms, succinate (Figure 2B) and hydrocortone phosphate (Figure 2C); N-ERD patients were hypersensitive to HCs and CPs (Figure 2D), which have no similar chemical structure. These results indicate that the mechanism of hypersensitivity to intravenous steroids is not dependent on the steroid structure itself. In previous reports, it was speculated that hypersensitivity to corticosteroid preparations could be mediated by an IgE-mediated mechanism because of the positive skin reactions in some cases (29–32). However, when intradermal testing of HCs and MPSLs was performed in five patients, only one patient tested positive for both drugs in this study, suggesting that a non-IgE mechanism is the primary cause; however, further investigation is needed to confirm this hypothesis.

Based on the results of this study, the use of rapid intravenous corticosteroids, especially succinate steroids, should be contraindicated in patients with N-ERD. However, in actual clinical practice, because intravenous steroids are mainly administered slowly by intravenous drip infusion, we conducted an additional retrospective study of medical records in which intravenous drip steroids were administered over a period of 2 h or more. N-ERD patients who received MPSLs 40–125 mg or dexamethasone sodium phosphate (DEXp) 1.65–6.6 mg IV over 2 h during asthma exacerbations were randomly selected for this study. The results showed that 2/55 (3.6%) patients had a mild cough after starting MPSLs infusion, which disappeared after switching to DEXp infusion. In contrast, no airway side effects were observed in the 31 N-ERD patients who received DEXp. These results suggest that a 2-h drip IV infusion of succinate corticosteroids is much safer than rapid IV infusion in N-ERD, and that drip IV infusion of a nonsuccinate corticosteroid such as DEXp may be the safest. This result was consistent with the findings of Dajani et al. (15).

Airway symptoms attributable to the succinate ester structure had two characteristics. First, they appeared earlier than symptoms caused by NSAIDs and were recognized within 5 min after intravenous injection; the onset of hypersensitivity symptoms in the N-ERD aspirin challenge study took 10–20 min or longer (25, 26, 33). This principle applies not only to oral aspirin studies but also to our proposed intravenous lysine-aspirin study (27, 28). The second is that the symptoms begin with a severe cough, which is not common in NSAID-induced symptoms. The mechanism of these two features is unknown, but the hypersensitivity due to the succinate ester structure may be a different mechanism than that of aspirin-induced asthma exacerbation. Aspirin desensitization was later performed on one of the patients studied in this study, but the hypersensitivity to HCs did not disappear. A similar phenomenon was also reported by Feigenbaum et al. (19).

The COX-1 inhibitory effect of succinate corticosteroids was examined in *in vitro* experiments using human platelets (34), but HCs did not inhibit COX-1 (data not shown). Based on these results, it was inferred that succinate corticosteroids cause hypersensitivity symptoms in N-ERD patients by mechanisms other than COX-1 inhibition.

In the present study, the frequency of hypersensitivity symptoms of HCs in N-ERD was higher than in three previous reports, with 9/11 (82%) cases showing a clear exacerbation of airway symptoms at 100–500 mg load. Reasons for this include the high loading dose (15, 16, 19) and the fact that the patients in the study were underweight. In the present study, hypersensitivity was induced in 36% of subjects at the 100 mg HCs provocation, whereas at the 500 mg load, hypersensitivity was induced in 76% of 100 mg negative subjects, indicating that hypersensitivity occurs in a dose-dependent manner.

HCs is rapidly degraded to HC and succinic acid within 5 min after intravenous injection, and blood concentrations of succinic acid increase rapidly (35). Succinic acid is an intermediate in the Krebs cycle, an endogenous substance that is constantly produced *in vivo* and accumulates inside and outside the cell under metabolic stress (36). Therefore, it is unlikely that normal low concentrations of succinic acid would cause hypersensitivity symptoms. However, immediately after intravenous administration of the corticosteroids succinic acid and CMs, it is inferred that the blood concentration of succinic acid rises unnaturally rapidly. The fact that slow drip infusion rarely causes hypersensitivity symptoms and negative skin tests suggest that, unlike IgE-mediated allergic mechanisms, only high concentrations of succinic acid are likely to induce hypersensitivity symptoms.

**Figure 3:**
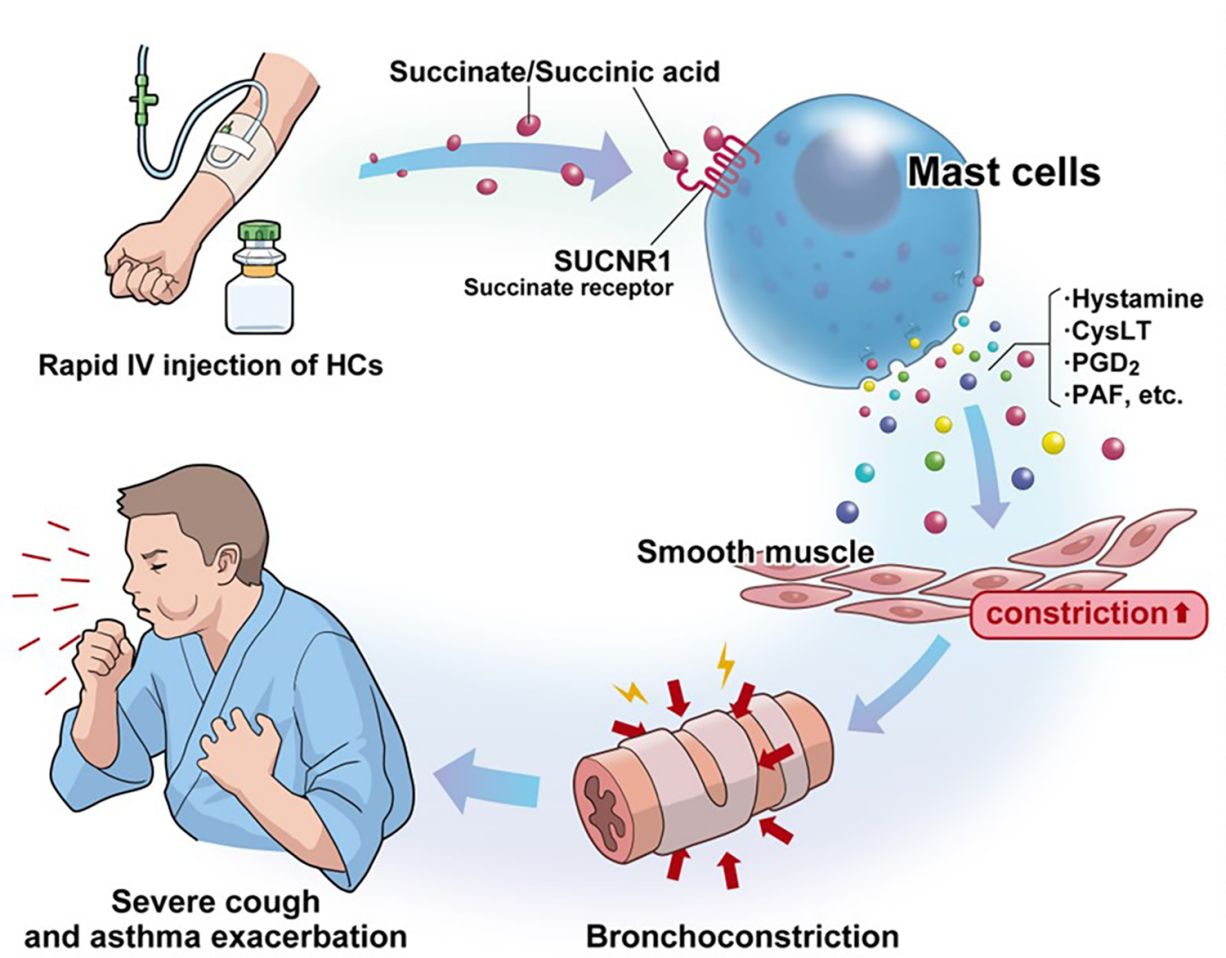
Hypothetical mechanism by which high concentrations of succinate/succinate ester cause hypersensitivity symptoms in N-ERD: persistent mast cell activation is a characteristic pathophysiology in N-ERD. Rapid intravenous infusion of drugs with succinate ester structures increases the blood concentration of succinate. Succinate/succinate ester stimulates succinate receptors (SUCNR1), which are highly expressed on human mast cells, resulting in a severe cough within 5 min of injection. N-ERD, nonsteroidal anti-inflammatory drug-exacerbated respiratory disease; HCs, hydrocortisone sodium succinate; SUCNR1, succinate receptor 1; CysLT, cysteinyl leukotrienes; PGD_2_, prostaglandin D_2_; PAF, platelet-activating factor.

In previous animal and *in vitro* models, succinic acid itself has been found to potentiate anaphylactic reactions and promote the release of histamine and SRS-A (37–39). Succinic acid has recently been shown to be involved in many immune and metabolic modulations via GPR91 receptors (40), and it has also been noted that succinic acid in the intestinal tract can exacerbate type 2 inflammation (41). The Karolinska Institute group recently published results showing that succinate receptor 1 (SUCNR1) is abundant in human mast cells and that succinate causes mast cell activation (36). Many reports have already shown that mast cell activation persists even during the stable phase as a characteristic pathology of N-ERD (42–48). N-ERD is more hypersensitive to adenosine inhalation, which stimulates mast cells (49), and the mast cell stabilizer cromolyn improves lung function only in stable adult asthmatics with N-ERD (50). Furthermore, omalizumab, an anti-IgE agent, has been demonstrated to significantly improve airway symptoms and aspirin sensitivity in N-ERD, in addition to inhibiting mast cell activation (48, 50–52). We hypothesized that when succinate ester preparations are rapidly administered to patients with N-ERD, their breakdown produces large amounts of succinate, which strongly activates mast cells via SUCNR1. This hypothesis is consistent with the course of a severe cough appearing within 5 min (Figure 3). However, evidence for this hypothesis is insufficient and further studies are needed.

The present study has several limitations. First, because the intravenous corticosteroid-induced study was performed during the stable phase, we have not been able to determine the correct threshold for HCs during asthma exacerbations. Second, the number of patients was small. Third, this study was a retrospective analysis of provocation records from the 1980s in Japanese patients only, not a randomized controlled trial with placebo. Fourth, the severity of asthma patients in this study was moderate to severe, which may have resulted in a low threshold of HCs.

In summary, the corticosteroid hypersensitivity in patients with N-ERD may not be due to the structure of the corticosteroid, but rather to the structure of the succinate ester. Furthermore, it was hypothesized that rapid activation of mast cells by succinate receptor stimulation is the mechanism of the hypersensitivity reaction; it should be emphasized that rapid intravenous administration of corticosteroids during attacks is dangerous in patients with N-ERD.

## Data Availability

The original contributions presented in the study are included in the article/Supplementary Material, further inquiries can be directed to the corresponding author.
